# Components of SurA Required for Outer Membrane Biogenesis in Uropathogenic *Escherichia coli*

**DOI:** 10.1371/journal.pone.0003359

**Published:** 2008-10-06

**Authors:** Kristin M. Watts, David A. Hunstad

**Affiliations:** 1 Department of Pediatrics, Washington University School of Medicine, St. Louis, Missouri, United States of America; 2 Department of Molecular Microbiology, Washington University School of Medicine, St. Louis, Missouri, United States of America; Theodor-Boveri-Institut fur Biowissenschaften, Wurzburg, Germany

## Abstract

**Background:**

SurA is a periplasmic peptidyl-prolyl isomerase (PPIase) and chaperone of *Escherichia coli* and other Gram-negative bacteria. In contrast to other PPIases, SurA appears to have a distinct role in chaperoning newly synthesized porins destined for insertion into the outer membrane. Previous studies have indicated that the chaperone activity of SurA rests in its “core module” (the N- plus C-terminal domains), based on *in vivo* envelope phenotypes and *in vitro* binding and protection of non-native substrates.

**Methodology/Principal Findings:**

In this study, we determined the components of SurA required for chaperone activity using *in vivo* phenotypes relevant to disease causation by uropathogenic *E. coli* (UPEC), namely membrane resistance to permeation by antimicrobials and maturation of the type 1 pilus usher FimD. FimD is a SurA-dependent, integral outer membrane protein through which heteropolymeric type 1 pili, which confer bladder epithelial binding and invasion capacity upon uropathogenic *E. coli*, are assembled and extruded. Consistent with prior results, the *in vivo* chaperone activity of SurA in UPEC rested primarily in the core module. However, the PPIase domains I and II were not expendable for wild-type resistance to novobiocin in broth culture. Steady-state levels of FimD were substantially restored in the UPEC *surA* mutant complemented with the SurA N- plus C-terminal domains. The addition of PPIase domain I augmented FimD maturation into the outer membrane, consistent with a model in which domain I enhances stability of and/or substrate binding by the core module.

**Conclusions/Significance:**

Our results confirm the core module of *E. coli* SurA as a potential target for novel anti-infective development.

## Introduction

Integrity of the outer membrane (OM) of Gram-negative bacteria relies on the coordinated expression, maturation, and insertion of lipopolysaccharide and a number of integral membrane proteins. A major subset of OM proteins (OMPs), existing in monomeric or multimeric forms, adopt pore structures upon their insertion into the membrane. Recent studies have informed a model for the process by which these porins traverse the periplasm and reach their destination in the OM. Nascent polypeptides destined for OM insertion enter the periplasm via the Sec translocon as the canonical signal sequence is cleaved. Hydrophobic portions of the primary sequence, which are common to integral OM proteins, might be expected to require protection by chaperones during transit through the periplasm. The protected polypeptides are delivered to an OM protein assembly complex anchored by BamA (also known as YaeT) [1]–[3], which coordinates the process of insertion through incompletely understood mechanisms.

Multiple lines of evidence implicate the periplasmic peptidyl-prolyl isomerase (PPIase) SurA in the chaperoning of β-barrel porins through the periplasm. At least three families of PPIases are encoded by *Escherichia coli* K-12; representative periplasmic proteins are the cyclophilin PpiA [4], the FK binding protein-like isomerase FkpA [5], and two parvulin domain-containing isomerases, SurA and PpiD [6]–[8]. These proteins feature in common one or more PPIase domains that catalyze the *in vitro* isomerization of proline bonds [9]. Though FkpA also exhibits chaperone activity [10], [11], SurA is uniquely positioned as a facilitator of periplasmic transit of nascent outer membrane porins. The relative lack of two major OMPs, OmpA [6] and LamB [6], [12], in *surA* mutants of *E. coli* K-12 was reported by two groups in 1996. More recently, we demonstrated that the pilus usher proteins FimD and PapC were SurA-dependent OMPs [13]. Mutation in *surA* results in accumulation of unfolded intermediates in the periplasm [12] and activation of the σ^E^ stress-response system [12], [14], which includes transcription of the periplasmic chaperone/protease *degP* [6]. More direct evidence of the involvement of SurA in OMP trafficking through the periplasm has been provided by Silhavy and colleagues. Mutations in *surA* were shown to be synthetically lethal with those in *degP* or in *skp*, which encodes a distinct and structurally unrelated periplasmic chaperone [15]. Subsequent studies in which SurA was depleted in a graded fashion showed that SurA was the primary chaperone responsible for OMP transit, while Skp and DegP likely can compensate to an extent when OMPs fall off the SurA pathway. Further, SurA was shown to interact directly with BamA *in vivo* [16].

The crystal structure of SurA from *E. coli* K-12, identical in primary sequence to that of other *E. coli* strains (including UPEC) and highly similar to those expressed by *Salmonella*, *Shigella*, and *Yersinia* [17], [18], was solved in 2002 [19]. The protein includes four distinct structural domains: an N-terminal domain with no obvious homology to other protein families, two parvulin-like PPIase domains (herein denoted I and II), and a short C-terminal domain. In the three-dimensional structure, the N and C-terminal domains together form a “core module” that is completed by a strand from PPIase domain I, while domain II extends away from this core module [19]. *In vitro*, the chaperone preferentially binds peptide sequences containing two aromatic residues separated by another amino acid (Ar-X-Ar), a motif that is over-represented in integral OM proteins of *E. coli* compared to proteins in other cellular compartments [20]–[22]. Finally, other studies have suggested that the chaperone activity of SurA localizes not to its two parvulin-like PPIase domains, but to its N-terminal substrate-binding domain. These studies relied on its interaction with non-native substrates, namely protection of citrate synthase from aggregation and binding to somatostatin [14], [23]. In this study, we aimed to investigate the components of SurA necessary for chaperone action in a pathogenic strain of *E. coli* and using chromosomally expressed, native SurA-dependent proteins. We interrogated SurA function using *in vivo* phenotypes relevant to *E. coli* uropathogenesis, namely resistance to membrane-impermeable antimicrobials and surface expression of the type 1 pilus usher FimD.

## Materials and Methods

### Bacterial strains and media

*E. coli* was grown in Luria-Bertani (LB) medium or Mueller-Hinton medium as indicated (Difco, Becton-Dickinson, Sparks, MD). UPEC strain UTI89 was recovered from the urine of a patient with cystitis [24]; C600 is a laboratory *E. coli* K-12 strain used for protein production. The UTI89 *surA* mutant was created by insertional disruption as described [25]. A panel of SurA domain constructs in the expression vector pQE30 was kindly provided by Dr. Susanne Behrens [14]. The coding region of each construct was amplified by high-fidelity PCR (Stratagene, La Jolla, CA) incorporating an XbaI site into the reverse primer. PCR products were digested with EcoRI and XbaI, and each resulting fragment was then ligated into the expression vector pTRC99 (GE Healthcare/Pharmacia, Piscataway, NJ). Empty vector (denoted pEV) and vector encoding full-length, native SurA (called pDH23) were included as controls where indicated. Expression was induced by addition of isopropyl β-d-1-thiogalactopyranoside (IPTG; Sigma, St. Louis, MO) at the indicated concentrations.

### Fractionation and Western immunoblotting

For periplasm preparation, cultures of the indicated strains grown in LB broth were harvested at mid-logarithmic phase, after IPTG induction during the last 40 min of growth. Cell pellets were resuspended in 20 mM Tris (pH 8.0) with 20% (w/v) sucrose; EDTA was added to 5 mM and lysozyme to 80 µg/mL, and the mixture was incubated on ice 20 min. MgCl_2_ was added to 25 mM and cell debris was pelleted by centrifugation. Supernatants (periplasms) were stored at 20°C until use. For preparation of outer membranes, overnight static cultures were back-diluted with LB broth to equivalent optical densities (at 600 nm), centrifuged and the weighed cell pellets resuspended in 10 mM Tris (pH 8.0) with 100 µg/mL each of DNase and RNase (Sigma, St. Louis, MO). The suspensions were lysed in a French pressure cell (14,000 psi) for two passages, and debris was removed by low-speed centrifugation. Membranes were collected by ultracentrifugation (33,000× *g* for 80 min) and pellets resuspended in 5 mL of 50 mM Tris (pH 8.0) with 1% N-laurylsarcosine (Sigma). After 1 h at RT with gentle rocking, ultracentrifugation was repeated, supernatants (representing inner membranes) were decanted and the pellets (outer membranes) were resuspended in Tris buffer. For detection of SurA variants, equal amounts of identically prepared periplasmic fractions were subjected to sodium dodecyl sulfate-polyacrylamide gel electrophoresis (SDS-PAGE) and transferred to polyvinylidene difluoride membranes (PVDF; Millipore, Billerica, MA). Full-length SurA with a 6-histidine tag was expressed in *E. coli* strain C600 and purified by metal-affinity chromatography. Antibodies directed against full-length SurA were raised in mice using standard Freund's adjuvant-based immunization and serum collection techniques, according to a protocol approved by the institutional Animal Studies Committee. Bound anti-SurA antibodies were recognized with alkaline phosphatase-conjugated anti-mouse IgG and visualized using BCIP/NBT substrate (both from Sigma). For FimD immunoblotting, outer membrane fractions were subjected to SDS-PAGE, transferred to PVDF membranes, and overlaid with mouse antiserum to FimD (MedImmune, Gaithersburg, MD). Bound anti-FimD antibodies were detected with peroxidase-conjugated anti-mouse IgG (Sigma) and visualized with CDP-Star substrate (Tropix Inc., Bedford, MA) and ECL Hyperfilm (GE Healthcare/Amersham, Pittsburgh, PA). For presentation, blots were scanned using an Epson 4470 scanner and band intensities quantified with ImageJ software (National Institutes of Health, Bethesda, MD). Data presented are representative of multiple independent experiments.

### Novobiocin growth assays

For disk diffusion assays, overnight cultures of the indicated strains in Mueller-Hinton broth (containing selective antibiotic) were subcultured, grown to equivalent optical densities, and swabbed in a lawn on freshly prepared Mueller-Hinton agar plates containing 0.01 mM IPTG. Filter paper disks containing 30 µg novobiocin (Becton-Dickinson) were placed on the agar, and the diameter of the zone of clearance was recorded after a second overnight incubation at 37°C. For bacterial growth curves, overnight cultures of the indicated strains were subcultured 1∶200 into fresh LB broth (with antibiotics and IPTG) in wells of a 24-well plate, then shaken at 37°C in a Synergy 2 multimode microplate reader (Bio-Tek, Winooski, VT) with absorbance readings at 600 nm recorded every 30 min.

### *In vitro* hemagglutination (HA), binding and invasion

Type 1 pilus-dependent hemagglutination of guinea pig erythrocytes (Colorado Serum Co., Denver, CO) was assayed in 96-well V-bottom plates as described previously (19). For binding and invasion experiments, cultured 5637 human bladder epithelial cells (ATCC HTB-9) were obtained from the American Type Culture Collection (Manassas, VA) and grown in RPMI 1640 medium (Gibco/Invitrogen, Grand Island, NY) supplemented with 10% fetal bovine serum (Sigma) at 37°C in a humidified atmosphere of 95% air and 5% CO_2_. Two days prior to assay, cells were detached with trypsin (0.05%) plus EDTA (0.02%), centrifuged, resuspended in fresh medium, and allocated to wells of sterile 24-well tissue culture plates. On the day of assay, confluent monolayers were washed once with sterile PBS, and fresh medium was applied prior to infection with 10^7^ CFU/mL of the indicated strains. Quantitation of cell-associated bacteria and invaded bacteria (via gentamicin protection) was performed as previously described [26].

### Statistical analysis

Two-tailed Student's T-tests were used for comparison of numerical data. For binding and invasion assays, relative binding and invasion by UPEC expressing SurA variants was reported as proportional to wild-type UPEC in each experiment, and the aggregate data were presented and statistically compared. A p value of less than 0.05 was considered statistically significant.

## Results and Discussion

### Expression of SurA domain variants in UPEC

**Figure 1:**
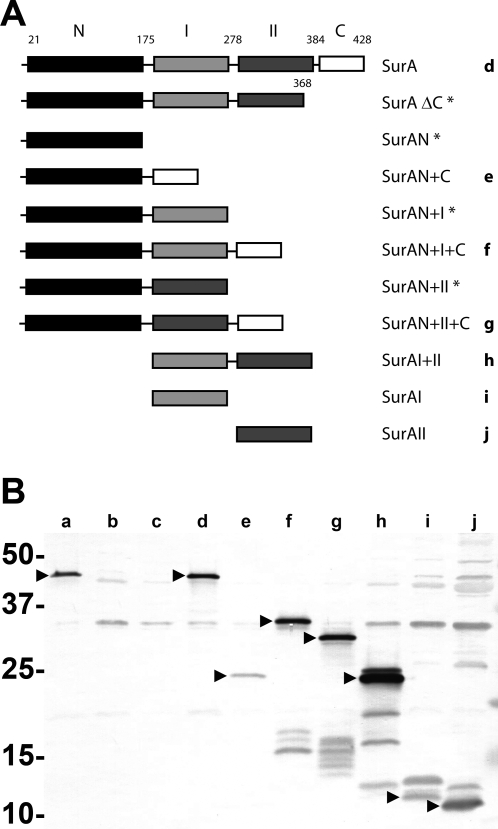
Complementation of the UPEC *surA* mutant with SurA domain constructs. (A) Schematic representation of the domains of mature periplasmic SurA, with amino acid residues (at top) numbered as in the synthesized polypeptide. Notation is adapted from reference 2. Constructs marked with an asterisk, containing the N-terminal but not the C-terminal domain, do not express stable protein variants ([ref. 14] and data not shown). Letters at right denote lane position in the blot in the lower panel. (B) Expression of domain variants in UPEC, as assessed by immunoblot of periplasmic extracts using mouse antiserum raised against full-length SurA with a 6-histidine tag. Approximate molecular masses are shown to the left. Lane a is from wild-type UTI89, b from the UTI89 *surA* mutant, and subsequent lanes from the UTI89 *surA* mutant complemented with plasmids expressing the following constructs (with predicted band sizes): c, empty vector; d, pDH23 (full-length SurA, 46kD); e, pSurAN+C (24 kD); f, pSurAN+I+C (34 kD); g, pSurAN+II+C (32 kD); h, pSurAI+II (22 kD); i, pSurAI (12 kD); j, pSurAII (11 kD). Bands representing expressed variants are denoted with arrowheads.

The set of domain constructs used in this study is shown in **Figure 1A**. For our studies, we excluded constructs that encoded the N-terminal domain but lacked the C-terminal domain, because the resulting polypeptides were previously found to be unstable ([14] and S. Behrens, personal communication). The remaining domain constructs were migrated to a different expression system and host strain than that earlier described [14]; therefore, we first evaluated expression of the domain variants in a uropathogenic strain of *E. coli*. Periplasms were prepared from UTI89 *surA*::*kan* complemented with full-length *surA* (on plasmid pDH23) or each of these domain constructs (induced with 0.01 mM IPTG). The periplasms were subjected to SDS-PAGE and immunoblotting using mouse antiserum raised against full-length SurA. Constructs containing the N- and C-terminal domains, with and without the PPIase domains (i.e., N+C, N+I+C, N+II+C, and full-length SurA) were all detectable at predicted sizes by Western blotting with mouse polyclonal antiserum raised against full-length SurA (**Figure 1B**). The combination of domains I+II was also detected readily; of these individual domains, domain II was more prominent than domain I on multiple blots. These results are generally consistent with those found in the constructs' original expression system in *E. coli* K-12 [14], with the exception that domain I appears less stable than domain II when expressed alone in the uropathogen. Steady-state periplasmic levels of the SurA variants with induction at higher IPTG concentrations (up to 0.1 mM) were not significantly different (data not shown).

### Membrane permeability and antibiotic resistance

**Figure 2:**
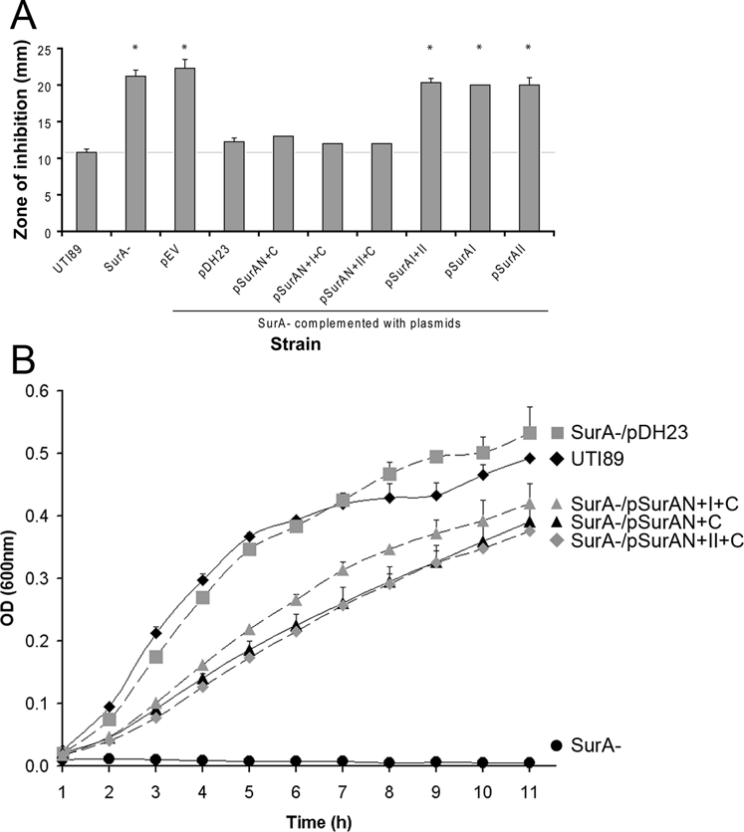
Restoration of novobiocin resistance in UTI89 by SurA variants. (A) Susceptibility to novobiocin is reported as the diameter of the zone of inhibition around 11-mm novobiocin disks after overnight incubation on Mueller-Hinton agar. Thus, wild-type UTI89 (leftmost bar) grows up to the disk edge (disk diameter indicated by gray line), and the *surA* mutant is susceptible to novobiocin under these conditions. Resistance is complemented by full-length SurA or the N+C domains. Domains I and/or II alone do not restore novobiocin resistance (*p<0.001 vs. UTI89); either domain I or II, when included with the N+C domains, bolsters resistance by a nonsignificant amount. Results represent the average of three separate experiments. (B) Growth of the indicated strains in LB broth containing 10 µg/mL novobiocin. The *surA* mutant is nonviable (black circles), and complementation with full-length SurA (on pDH23; gray squares) restores growth equivalent to wild-type UTI89 (black diamonds). Under these conditions, the N+C variant incompletely rescues novobiocin resistance (black triangles), and a small but significant contribution of domain I to novobiocin resistance is revealed (gray triangles; p<0.05 versus N+C from 2 to 8 h). Absent growth of the *surA* mutant complemented with empty vector, pSurAI, pSurAII, or pSurAII was indistinguishable from the *surA* mutant itself; these curves were omitted from the graph for clarity.

To determine general effects on the membrane of UPEC during complementation of the *surA* mutant with the SurA domain constructs, we assessed growth in the presence of novobiocin, a hydrophobic aminocoumarin antibiotic and inhibitor of DNA gyrase [27] that normally does not penetrate the Gram-negative cell envelope. We have previously demonstrated that lack of SurA imparts susceptibility to novobiocin in *E. coli* K-12 [13]. As measured by disk diffusion with 11-mm, 30-µg novobiocin filter paper disks, growth inhibition of the UTI89 *surA* mutant was significantly greater than that of wild-type UTI89 (**Figure 2A**). Complementation of the mutant with constructs containing the N- plus C-terminal domains of SurA restored wild-type resistance; inclusion of domain I or II did not significantly alter this phenotype. In contrast, domains I or II alone or in combination, though stably expressed, did not restore novobiocin resistance (p<0.001 versus wild type). We next proceeded to examine novobiocin susceptibility in an alternative way, namely the growth of the same strains in LB broth culture containing 10 µg/mL of novobiocin. Under these conditions, the N and C-terminal domains together substantially restored novobiocin resistance to the *surA* mutant (**Figure 2B**), and addition of domain I (but not domain II) augmented growth (p<0.05). The broth culture experiments were therefore more sensitive in detecting a role for domain I; such a role might relate either to stability of the SurA “core module” (as suggested by **Figure 1B**) or perhaps to aspects of SurA function, such as substrate binding.

### Type 1 pilus-dependent phenotypes of UPEC

**Figure 3:**
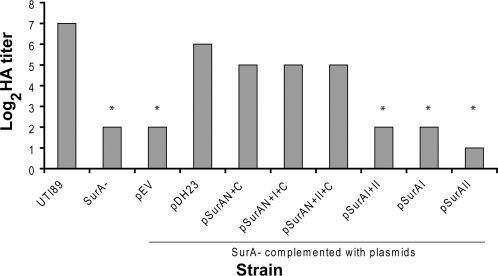
Hemagglutination (HA) by SurA domain-complemented UTI89. Uniform suspensions of the indicated strains were mixed with a series of two-fold dilutions of guinea pig erythrocytes, and the overnight HA titer is shown. HA is substantially complemented by full-length SurA or any of the N+C-containing variants, while domains I and II (alone or in combination) fail to complement the HA defect of the *surA* mutant (*p<0.01 versus WT). Results are representative of three separate experiments.

The capacity of UPEC to bind and invade the bladder epithelium is conferred by type 1 pili and, more specifically, by the mannose-sensitive type 1 tip adhesin FimH [26]. Our recent work demonstrated that inactivation of *surA* in *E. coli* K-12 or in UTI89 resulted in significantly decreased piliation, corresponding with a decrement in steady-state levels of the type 1 pilus usher FimD in the OM [13], [28]. We applied similar analyses to the UTI89 *surA* mutant complemented with SurA domain variants. First we assayed the agglutination of guinea pig erythrocytes, an *in vitro* phenotype dependent on FimH, by UTI89 expressing the SurA variants. Consistent with our previous results, loss of SurA led to a significant reduction in hemagglutination (HA) titer (**Figure 3**), and the residual HA was inhibited by the addition of 2% methyl-α-d-mannopyranoside (data not shown). Complementation with full-length SurA or with the N- plus C-terminal domains substantially restored the HA titer. Inclusion of domain I or II did not provide an additive effect on the HA titer, and domains I and/or II alone provided no complementation. For N+C, N+I+C, and N+II+C, increases in the concentration of inducer (to 0.1 mM IPTG) caused piliation levels and HA titers to decrease (data not shown), suggesting that accumulation of imperfect variants in the periplasm or occupation of other chaperone or periplasmic stress-response systems had a detrimental effect on pilus assembly and presentation.

**Figure 4:**
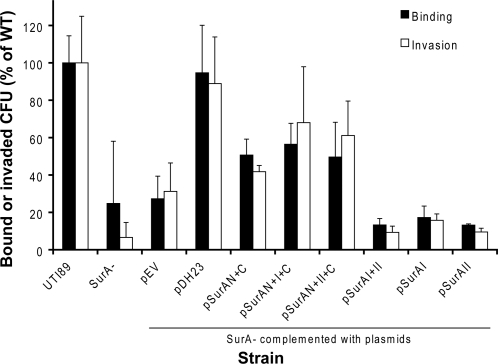
Binding and invasion of cultured bladder epithelial cells by SurA domain-complemented UTI89. Relative binding and invasion capacities are shown as a proportion of wild-type UTI89 binding and invasion; results for each strain represent the aggregate of at least three independent experiments. Binding and invasion are significantly reduced in the *surA* mutant. The three variants containing the N+C domains substantially restored binding and invasion (p<0.04 versus *surA* mutant), but below wild-type levels (p<0.0001 versus WT for binding; p<0.002 for invasion). Addition of domain I or II to N+C was associated with a significant increase in invasion capacity (p<0.01). The three SurA variants containing only domains I and/or II failed to restore these type 1 pilus-dependent functions (p<0.0001 versus WT for both binding and invasion).

To more specifically investigate the relevant type 1-dependent functions of the SurA variant strains, we next assayed the binding and invasion of cultured human bladder epithelial cells by UPEC expressing the SurA variants. Confluent monolayers of 5637 human bladder epithelial cells were infected with each strain; binding was evaluated after washing and homogenization of the monolayers, and invasion was assessed by gentamicin protection [29]. Consistent with our prior results [28], there was a sharp decrement in epithelial binding by the *surA* mutant when compared with wild-type UTI89, and this defect was complemented by provision of full-length *surA* in trans (**Figure 4**). Domain constructs encoding both the N- and C-terminal domains of SurA substantially but incompletely restored both binding and invasion (p<0.04 for binding and invasion versus the *surA* mutant, either alone or with empty vector; p<0.0001 for binding and p<0.002 for invasion versus wild type). The addition of either domain I or II to the N- plus C-terminal domains had no significant impact on binding but conferred significant increases in invasion (p<0.01 for each comparison versus N+C). Binding and invasion by UTI89 expressing SurA variants including only domains I and/or II (without the core module) were indistinguishable from the *surA* mutant.

### Steady-state production of FimD

**Figure 5:**
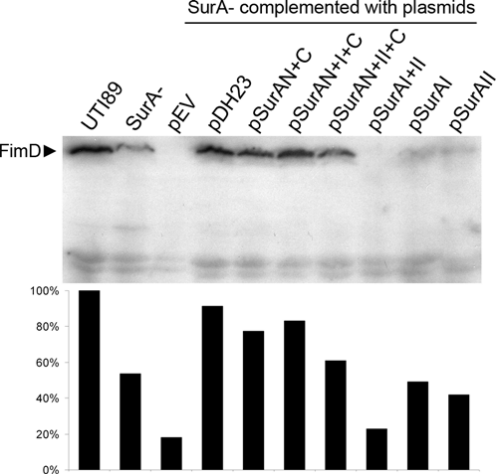
Usher levels in the OM. Steady-state levels of FimD in total membrane extracts from the indicated strains were determined by SDS-PAGE followed by Western immunoblotting (upper panel) using a mouse antiserum to FimD. Quantitation of band intensity is shown in the lower panel, expressed relative to wild type. Consistent with previous results, FimD presence is sharply reduced in the *surA* mutant. FimD is restored upon complementation with full-length SurA. The N+C variant substantially restores FimD stability, and addition of domain I (but not domain II) augments this phenotype. Domains I and/or II alone provide minimal chaperone function. Cross-reacting bands are included in the figure to demonstrate overall protein loading.

Our previous data suggested that the type 1 pilus usher FimD is a SurA-dependent OM protein, and that failed maturation of this usher underlies defective piliation in *surA* mutants. Therefore, we examined the steady-state levels of FimD in UTI89 and the *surA* mutant expressing the SurA domain variants by Western blotting of outer membranes harvested from these strains. Consistent with prior results, disruption of *surA* led to a notable decrement in the presentation of FimD, and this was restored by complementation with full-length SurA (**Figure 5**). Mirroring the invasion data, domains N+C substantially restored FimD presence in the OM, and addition of domain I slightly augmented FimD levels. The PPIase domains alone contributed no support of FimD maturation in the OM. This experiment provides further evidence that the defect in type 1 piliation of *surA* mutants is due to failed maturation of FimD. In addition, our combined studies of the relationship between SurA and the type 1 pilus assembly system indicate that pilus production in UPEC relies primarily on activity of the core module of SurA and suggest a contribution from the PPIase domain(s), particularly domain I. Finally, we conclude that type 1 piliation and pilus-dependent functions in UPEC are proportional to the amount of usher present in the OM, suggesting that usher maturation might represent a means by which the bacterial cell can regulate the presentation of pili under different conditions.

Bacterial systems supporting the maturation, assembly, and insertion of outer membrane proteins are of critical importance to membrane integrity and function, including filtering, permeability, and secretion. In recent years much has been learned about the trafficking and assembly of porins into the OM. SurA appears to key the periplasmic transit of nascent, monomeric OMP species, delivering these to the OMP assembly complex anchored by BamA, itself an OMP. Indeed, recent data demonstrate a direct biochemical interaction of SurA with BamA *in vivo* [16]. Putative OMP sequences targeted for binding by SurA have been identified [20], [22], and the present study augments earlier findings regarding the portions of SurA critical for its chaperoning of OMPs [14], [23], [30]. Despite these advances, the spectrum of SurA-dependent OMPs is not precisely defined. While structural details of SurA interaction with model peptides have recently been published [30], similar demonstration of its binding to one or more *in vivo* OMP substrates is needed for a more complete understanding of its mechanism of action. Our ongoing studies aim to delineate the range of SurA-dependent proteins, to demonstrate direct SurA-substrate interactions, and to interrogate the structural details of the chaperone-substrate relationship.

Knowledge at a molecular level of the mechanisms of SurA function will also inform the development of small-molecule inhibitors of this important and conserved chaperone. Of primary interest, such an inhibitor might prove a valuable anti-virulence compound against Gram-negative pathogens. As a primary example, SurA provides pleiotropic support to virulence of uropathogenic *E. coli*. We have shown here that SurA keys type 1 piliation, a primary determinant in cystitis; but SurA-dependent proteins also support the intracellular phenotypes of UPEC ([28] and unpublished data) and the local suppression of epithelial proinflammatory cytokines [25]. Beyond *E. coli*, SurA is conserved in other Gram-negative pathogens, such as *Salmonella*, *Yersinia*, and *Shigella*; and a *Salmonella surA* mutant was attenuated after oral inoculation in mice [31]. In the laboratory, a genetic method for incremental control of *surA* expression (and thus SurA function) has recently been demonstrated [16]. However, an available inhibitor would simplify this control, offering broad potential applications in the study of Gram-negative envelope biology and outer membrane biogenesis.
